# Analysis of preoperative blood platelet parameters in terms of diversity of epithelial ovarian cancer: Erratum

**DOI:** 10.1097/MD.0000000000010835

**Published:** 2018-05-18

In the article, “Analysis of preoperative blood platelet parameters in terms of diversity of epithelial ovarian cancer”,^[1]^ which appeared in Volume 97, Issue 12 of *Medicine*, the author list should appear as:

Katarzyna Bednarska, PhD, Ewa Król, MScb, Ewa Głowacka, PhD, Hanna Romanowicz, PhD, Krzysztof Szyłło, Professor, Magdalena Klink, Professor, Zofia Sułowska, PhD, Marek Nowak, Professor
